# Differential expression patterns of glycosphingolipids and C‐type lectin receptors on immune cells in absence of functional regulatory T cells

**DOI:** 10.1002/iid3.334

**Published:** 2020-08-01

**Authors:** Adan C. Jirmo, Charlotte Rossdam, Ruth Grychtol, Christine Happle, Rita Gerardy‐Schahn, Falk F. R. Buettner, Gesine Hansen

**Affiliations:** ^1^ Department of Pediatric Pneumology, Allergology and Neonatology Hannover Medical School Hannover Germany; ^2^ Biomedical Research in Endstage and Obstructive Lung Disease Hannover (BREATH) German Center for Lung Research (DZL) Hannover Germany; ^3^ Institute of Clinical Biochemistry Hannover Medical School Hannover Germany; ^4^ Excellence Cluster RESIST (EXC 2155) Hannover Medical School Hannover Germany

**Keywords:** CD4+ T cells, dendritic cells, FOXP3‐deficient, glycosphingolipids

## Abstract

**Background:**

Glycosylation is a common and complex type of protein posttranslational modification. Altered glycosylation of immunoglobulins in autoimmune diseases has led to the “altered glycan hypothesis” postulating existence of a unique glycan signature on immune cells and extracellular proteins characterized by site‐specific relative abundances of individual glycan structures and glycosylation patterns. However, it is not clear how glycosylation on leukocyte subpopulations differ between states of health or inflammation.

**Hypothesis:**

Glycosphingolipid patterns on immune cells of forkhead‐box‐P3‐deficient scurfy mice differs from those on wild‐type immune cells.

**Methods:**

T cells and dendritic cells were isolated from spleens of either wild‐type or age‐matched scurfy mice. Glycosphingolipids of CD4^+^ T cells and splenic dendritic cells from wild‐type and scurfy mice were then analyzed by multiplexed capillary gel electrophoresis coupled to laser‐induced fluorescence detection (xCGE‐LIF). In addition, flow cytometry and ChipCytometry were used to access expression patterns of various C‐type lectin receptors on antigen‐presenting cells from various organs of both wild‐type and scurfy mice.

**Results:**

We, hereby report differential expression of glycosphingolipids in health and under inflammatory conditions as reflected in wild‐type and scurfy mice. Furthermore, we observed that the absence of functional regulatory T cells correlated with elevated expression of CLEC‐7A and CD205 but a reduction in levels of CLEC12A and CD206 on antigen‐presenting cells.

**Conclusion:**

We hereby show that the absence of functional regulatory T cells affects expression pattern and quantities of glycosphingolipids on immune cells. Thus, glycosphingolipids could serve as biomarkers for mapping genetical and homeostatic perturbances such as those resulting from a diseased condition.

## Graphical abstract

(a) Attenuated levels of glycosphingolipids in splenic dendritic cells in forkhead‐box‐P3 (FOXP3)‐deficient scurfy mice lacking functional regulatory T cells. (b) Glycosphingolipids patterns on CD4+ T cells are not affected in absence of FOXP3. (c) Expression patterns and quantities of glycosphingolipids on immune cells could serve as signatures to map inflammation and homeostatic conditions in health or as biomarkers for identifying homeostatic perturbances.

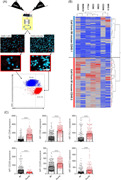

**Abbreviations**
AM: alveolar macrophages
DC: dendritic cell
FOXP‐3: forkhead‐box‐P3
T regs: regulatory T cells
xCGE‐LIF: multiplexed capillary gel electrophoresis coupled to laser‐induced fluorescence

## 1 INTRODUCTION

The glycocalyx is a dense array of glycans on surfaces of eukaryotic cells and together with proteins, nucleic acids, and lipids constitute one of the essential building blocks of a cell. As a result of glycosylation, a highly diverse repertoire of cellular glycans are attached either to proteins or lipids forming glycoproteins or glycolipids, respectively^1^ and thereby, influence or modulate individual proteins or lipids functions as key signaling moieties or in cell‐cell interactions.^2^

As such, several studies have validated not only the enormous structural and functional diversity of glycans but also their indispensable role in a variety of biological processes, including those that are fundamental for the development and homeostasis of the immune response.^1^, ^3^, ^4^, ^5^ Immune responses such as activation, differentiation, and homing have been shown to be accompanied by a programmed remodeling of cell‐surface glycans driven by glycosyltransferases and glycosidases.^6^, ^7^, ^8^ Both in humans and mice, the biological significance of the glycome is revealed in diseases caused by glycosylation defects.^9^, ^10^ Indeed, studies employing a mouse deficient of β^1^, ^2^, ^3^, ^4^, ^5^, ^6^ branching of tri‐ and tetra‐antennary complex N‐glycans have shown that the absence of this galectin‐3 ligand results in enhanced T cell receptor (TCR)‐mediated signaling and induced “hyper‐T_H_1” responses and greater susceptibility to autoimmune disease due to restricted TCR aggregation by binding to galectins in the immunological synapse.^8^, ^11^ Apart from N‐glycans, O‐glycans have been shown to regulate immune system homeostasis since differential sialylation of cell‐surface glycoproteins are reported to be capable of serving as an “on‐off” switch that controls decisions between immune cell responsiveness and tolerance.^11^ In addition, binding of the nonpolymorphic MHC class I‐like molecule CD1d expressed on antigen‐presenting cells by glycolipids activates natural Killer T cells (NKT) cells and has been shown to have modulatory effects on subsequent polarization of NKT cells or disease outcomes as in collagen‐induced arthritis in mice^12^, ^13^ and, can be used as adjuvants for humoral immune responses.^14^, ^15^

However, although the importance of glycosylation in the modulation of immune responses and homeostasis has been extensively appreciated, less is known regarding the nature of glycosylation patterns and in particular glycolipids on immune cells in inflammatory conditions. This is in part due to the fact that it is not easy to predict glycosylation patterns based only on a set of glycosyltransferases and glycosidases, since different cells have been shown to glycosylate the same protein backbone differently.^2^ Thus, we think, a contextual approach where underlying cellular differences are known could provide a valuable understanding of glycome patterns which could be used to delineate glycosylation signatures in health and disease. To address this question, we used forkhead‐box‐P3 (FOXP3)‐deficient scurfy (Sf) mice as a model of uncontrolled inflammation due to a lack of regulatory T cells (T regs). This model revealed that the lack of functional T regs changed levels and patterns of glycosphingolipids present on the surface of dendritic cells (DCs) but not on CD4^+^ T cells. Finally, we analyzed the surface expression of some C‐type lectin receptors (CTLRs) to understand if there is any difference in their expression in wild‐type (Wt) as compared with the inflamed condition as observed in Sf mice.

## 2 MATERIAL AND METHODS

Mice: FOXP3^+/−^ heterozygous females (B6.Cg‐Foxp3^sf^/J), nonaffected inbred males, and congenic CD45.1 mice, all with C57BL/6J genetic background, were originally purchased from The Jackson Laboratory (Bar Harbor, Maine). Male affected Sf mice and healthy littermate control mice (Wt) were analyzed at 3 weeks of age. Mice were housed in the animal facility of Hannover Medical School under specific pathogen‐free conditions.

Ethics statement: All experiments were approved by the local animal welfare committee of Lower Saxony State Office for Consumer Protection and Food Safety and performed strictly according to their guidelines.

### 2.1 Isolation of cells, chip cytometry, and flow cytometry

Both splenic CD4^+^ T cells and CD11c^+^ DCs were isolated from 21‐days‐old mice using commercially available Milteny Kits according to the manufacturer's protocol (Miltenyi Biotec, Bergisch Gladbach, Germany). Peritoneal macrophages were harvested from peritoneal lavage fluid by flushing the peritoneal cavity of either CD45.1 congenic Wt mice or CD45.2 Sf mice with 3 to 4 × 1 mL of cold sterile Hank's balanced salt solution (Sigma‐Aldrich, St. Louis, MO) as already described in our previous work.^16^ Peritoneal lavage cells were centrifuged and counted with Cedex HiRes automated cell analyser (Roche, Basel, Switzerland), density adjusted and loaded onto microfluidic ZellSafe chips (Zellkraftwerk GmbH, Leipzig, Germany) for further iterative chip‐based imaging cytometry as described previously.^17^, ^18^ Briefly, equal numbers of CD45.1 and CD45.2 peritoneal macrophages were loaded on a single ZellSafe chip and characterized using the following antibodies: MHC‐II, CD80, CD86, PD‐L1, CD206, CD205, and MARCO, all Phycoerithrin (PE) conjugated. Flow cytometry‐based phenotypic analysis of DCs and alveolar macrophages (AMs) in the lungs were performed using CD11c fluorescein isothiocyanate, MHC‐II Pacific Blue, Siglec‐F Allophycocyanine (APC)‐Cy7, Clec12A PE, and dendritic cell‐associated C‐type lectin‐1 (Dectin‐1) APC conjugated antibodies.

### 2.2 Glycosphingolipid extraction from splenic CD4^+^ T cells and CD11c^+^ DCs and analysis by multiplexed capillary gel electrophoresis coupled to laser‐induced fluorescence detection

Glycosphingolipids from cells were extracted and the β‐glycosylic linkage between the glycan and the ceramide of glycosphingolipids was enzymatically cleaved to release the respective glycan head groups for further chemical modification with a fluorescent label as previously described.^17^ Briefly, glycosphingolipids were prepared from cells via repeated chloroform/methanol extraction and subsequent purification using a Chromabond C_18_ ec polypropylene column (Macherey‐Nagel, Düren, Germany). The extracted glycolipids were incubated with LudgerZyme Ceramide Glycanase (Ludger, Oxfordshire, UK) derived from the leech *Hirudo medicinalis*, in LudgerZyme Ceramide Glycanase RXN buffer for 24 hours at 37°C. Obtained glycans were fluorescently labeled with 8‐aminopyrene‐1,3,6‐trisulfonic acid trisodium salt (Merck) and multiplexed capillary gel electrophoresis coupled to laser‐induced fluorescence detection (xCGE‐LIF) was performed as already described.^17^ The migration time range of 10 to 350 migration time unit (MTU) of the obtained xCGE‐LIF electropherograms was applied for further data processing using CorelDRAW 2017. To quantify intensities of various peaks, the heights of all peaks in the range of 10 to 350 MTUs were summed up and the height of individual peaks was calculated as percentage referring to the summarized peaks heights, resulting in relative signal intensities.

### 2.3 Statistical analysis

GraphPad Prism software version 6 was used for statistical analysis (GraphPad Software, La Jolla, CA) and Student *t* test was used to determine the statistical significance of differences between Wt and Sf mice. *P < .05, **P < .01, ***P < .001, and ****P < .0001 were considered as significant.

## 3 RESULTS

### 3.1 Lack of functional regulatory T cells associates with reduced glycosphingolipid levels in DCs but not in CD4^+^ T cells

Previous studies have shown that inflammations modify glycosylation of glycolipids and glycoproteins.^18^, ^19^ The presence of effector T cell populations with altered glycosylation have been described in autoinflammatory conditions such as systemic lupus erythematosus.^20^

**Figure 1:**
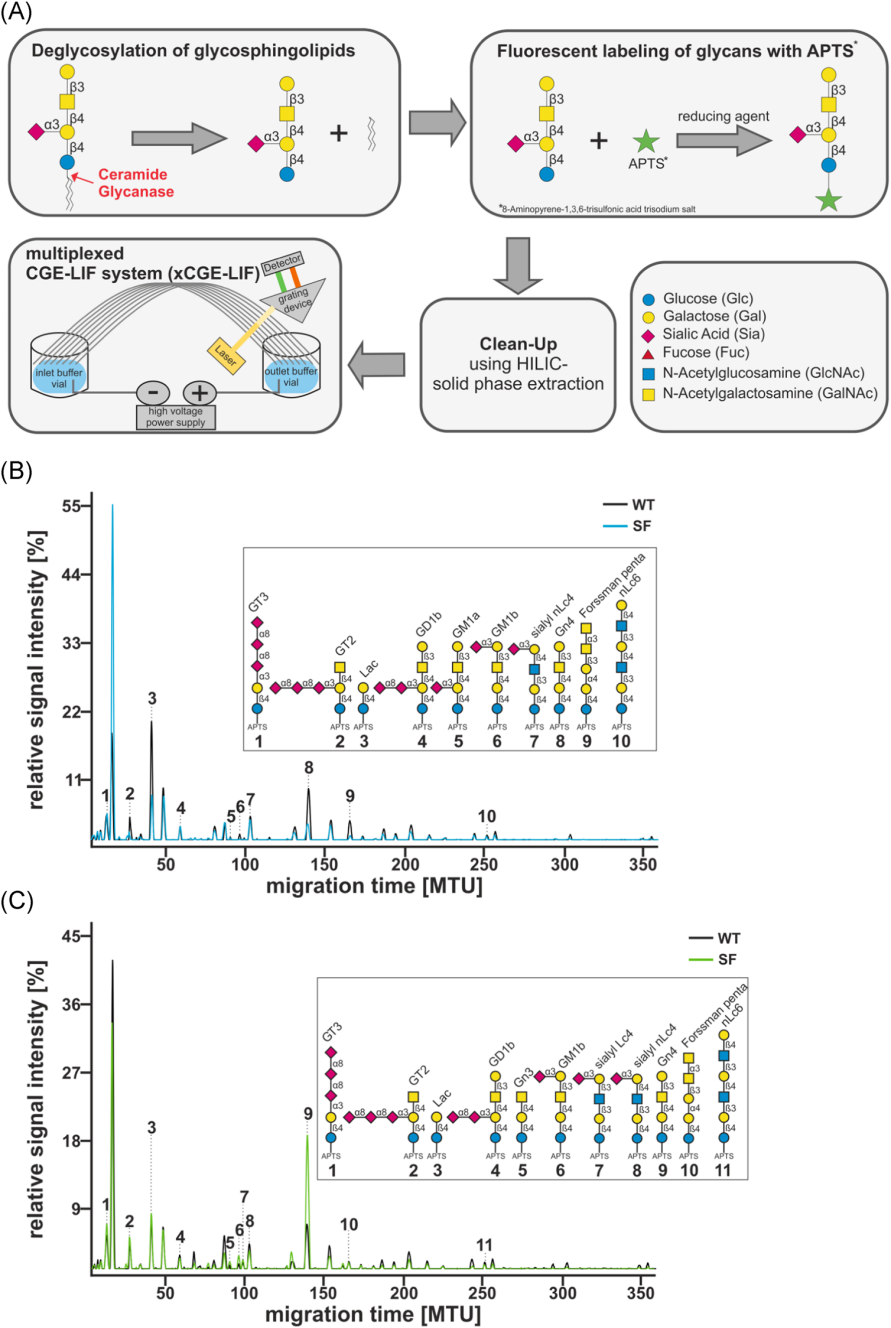
Analysis of glycosphingolipid glycosylation patterns on splenic DCs and CD4^**+**^ T cells: Schematic representation of glycosphingolipid glycosylation analysis (A). Representative electropherograms from splenic DCs (B) and CD4^+^ T cells (C) showing relative signal intensities of glycosphingolipid‐derived glycans. Migration time units (MTU) are glycan specific and by matching to our migration time database^17^ numerous peaks could be assigned to specific glycan structures. DCs, dendritic cells; xCGE‐LIF, multiplexed capillary gel electrophoresis coupled to laser‐induced fluorescence detection

**Figure 2:**
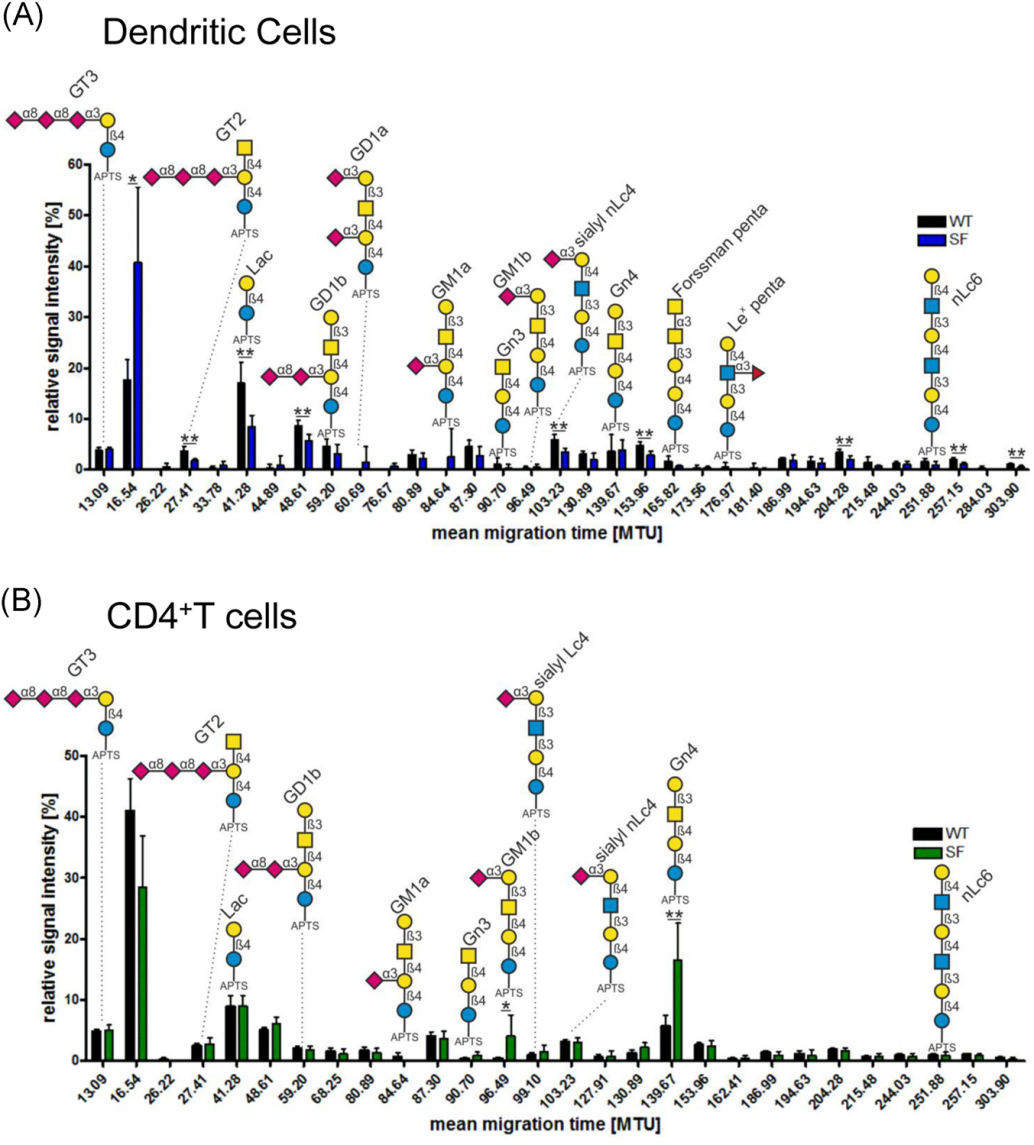
Analysis of glycosphingolipid‐derived glycans of splenic DCs and CD4^**+**^ T cells reveals alterations caused by the absence of functional T regs cells. DCs and CD4^+^ T cells were enriched from the spleens of both Wt and Sf mice and glycosphingolipid glycosylation was assessed by xCGE‐LIF (A and B) respectively. The error bars represent ±SEM and *P < .05, **P < .01, and ***P < .001 in two independent experiments with n = 5‐6 animals/group. DCs, dendritic cells; SEM, standard error mean; xCGE‐LIF, multiplexed capillary gel electrophoresis coupled to laser‐induced fluorescence detection

**Figure 3:**
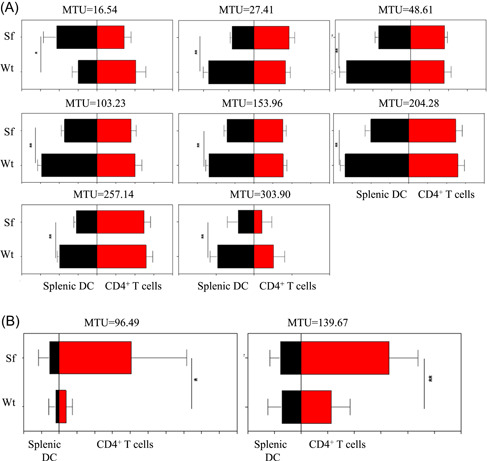
Different glycolipids are attenuated on splenic DCs and CD4^**+**^ T cells in the absence of functional T regs cells. Expression intensities of gylcosphingolipids on splenic DCs (A) and CD4^+^ T cells (B) showing a direct comparison of the same glycosphingolipid‐derived glycans on both DCs and CD4^+^ T cells of Wt and Sf mice. The error bars represent ±SEM and *P < .05, **P < .01, and ***P < .001 in two independent experiments with n = 5‐6 animals/group. DCs, dendritic cells; SEM, standard error mean; T regs, regulatory T cells

To understand how levels of glycosphingolipids in an inflammatory condition differ from noninflammatory steady‐state conditions, we enriched dendritic cells and CD4^+^ T cells from either Wt mice or Sf mice and analyzed glycosphingolipid glycosylation using a recently published approach by xCGE‐LIF^17^ (Figure 1A). This analysis revealed more than 30 and 28 baseline separated peaks from splenic DCs (Figure 1B) and splenic CD4^+^ T cells (Figure 1C), respectively. Based on our previously established migration time database,^17^ about onethird of these peaks could be assigned to distinct glycan structures (Figure 1B,C). Since glycosylation patterns have been shown to be influenced both by genetic and environmental factors such as cytokine milieu,^21^ we hypothesized that glycosphingolipid glycosylation patterns differ in DCs and CD4^+^ T cells isolated from Sf and Wt mice. Indeed, analysis of glycosphingolipid glycosylation on DCs revealed significantly reduced levels of the glycosphingolipids GT2, lactosylceramide (Lac‐Cer), and sialyl neolactotetraosyl ceramide (sialyl nLc4‐Cer) and other so far unassigned structures migrating at MTUs of 48.61, 153.69, 204.28, 257.15, and 303.90 in Sf mice as compared with those of Wt animals (Figure 2A). On the other hand, we observed significant upregulation of the gangliosides GM1b and Gn4 on CD4^+^ T cells isolated from Sf mice compared with T cells or splenic DCs from Wt animals (Figure 2B). Interestingly, none of the glycosphingolipid‐derived glycans that were significantly differentially expressed in Wt or Sf DCs were differentially expressed in CD4^+^ T cells of either murine strain and vice versa (Figure 3A,B).

### 3.2 Influence of functional T regs on activation of peritoneal macrophages and their expression of C‐Type lectins

**Figure 4:**
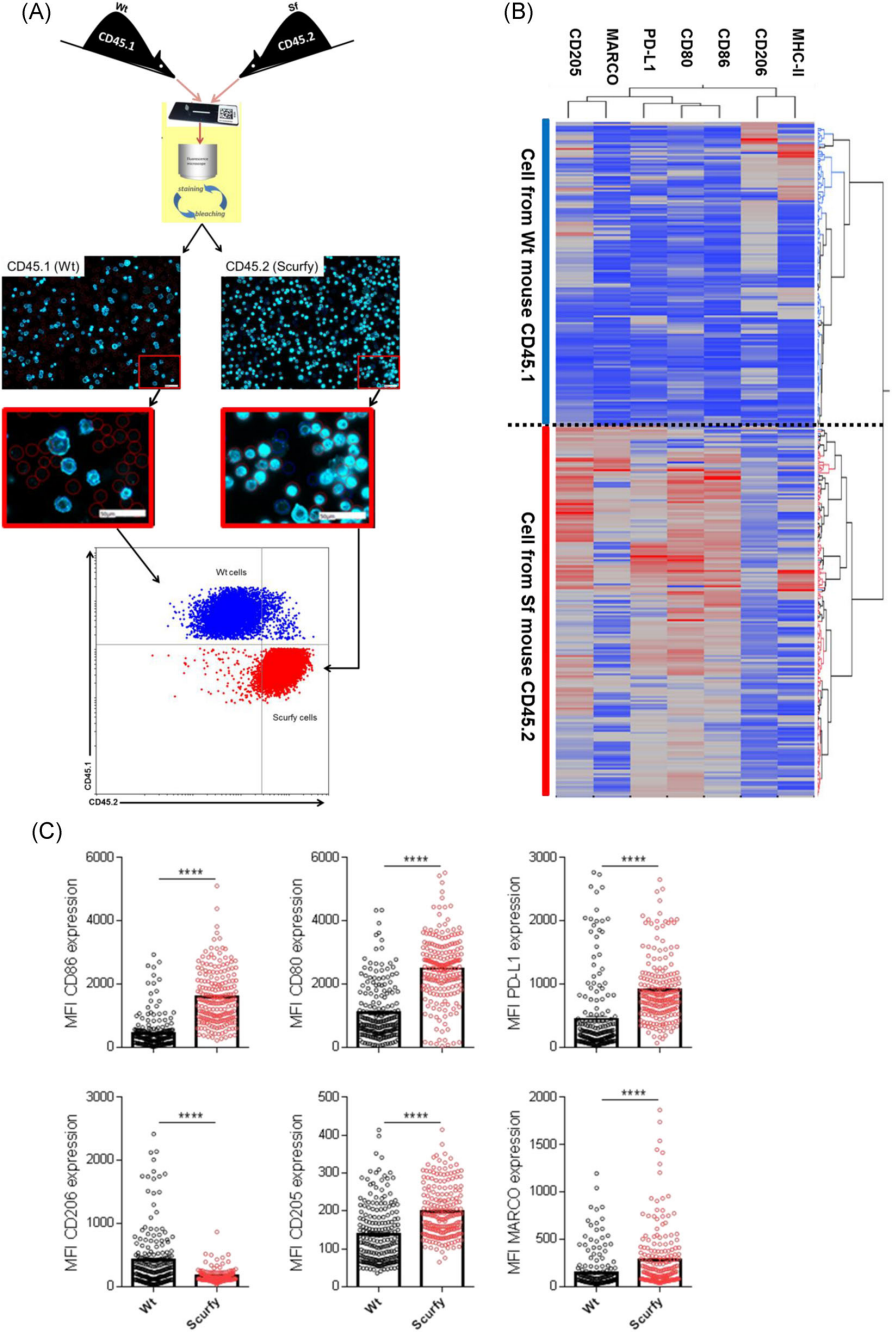
Enhanced activation of peritoneal macrophages in Scurfy mice compared with Wt mice: Peritoneal macrophages isolated from either CD45.1 congenic Wt mouse or CD45.2 congenic Sf mouse were simultaneously loaded on a single microfluidic chip for analysis using Chip Cytometry (A). Heatmap showing single cell expression pattern of MHC‐II, CD206, CD86, CD80, PD‐L1, MARCO, and CD205 by both Wt and Sf peritoneal macrophages (B). Quantification of various markers on single cells of either Wt or Sf mouse origin (C). The error bars represent ± SEM and ***P < .001 on 200 cells of either Wt or Sf origin randomly picked and analyzed for each marker. SEM, standard error mean; Wt, wild‐type

Lectins are a huge group of carbohydrate‐binding proteins, some of which serve as receptors specialized in the recognition of cell‐surface glycans.^1^ Molecular recognitions of glycans by lectins have been shown to mediate complex immunological cell‐to‐cell interactions, T cell homeostasis, inflammation as well as both innate and adaptive immune responses.^22^, ^23^ CTLRs have been shown to recognize carbohydrates leading to their internalization and presentation to effector cells such as T cells.^24^ Having observed differential expression in levels of various glycosphingolipids on the surface of splenic DCs from Wt and Sf mice, we speculated differences also in the expression of carbohydrate‐binding molecules such as CTLRs on myeloid cells between Wt and Sf mice in our study. This purely observational characterization was driven by the hypothesis that probably inflammatory cues as observed in Sf mice could affect CTLRs expression on myeloid cells such as macrophages and DCs and thereby be the cause of observed glycolipid patterns. Interactions of c‐type lectins on immature antigen‐presenting cells with glycosylated structures have been proven to downmodulate T cell antigen receptor‐mediated signaling and even suppression of chronic inflammation and autoimmunity in a model of T cell activation and homeostatic regulation.^23^ Consequently, in some studies, lectins have been used to suppress chronic inflammation in autoimmunity models such as in diabetes and autoimmune retinal disease through skewing the balance of immune response toward a Th2 cytokine profile^25^, ^26^, ^27^, ^28^ after binding their cognate glycan partners. However, information regarding expression levels of glycosphingolipids and lectins in inflammation such as those caused by lack of T reg cells as depicted in scurfy mice is rare. Thus, to analyse glycan‐binding molecules on antigen‐presenting cells, we enriched for peritoneal macrophages from Wt and Sf mice and used chip cytometry for single cell‐based analyses (Figure 4A). Applying CD45.1 and CD45.2 congenic markers, we separated the Wt cells from the Sf cells (Figure 4A) and subsequently analyzed markers associated with activation (CD86, CD80, and PD‐L1) as well as C‐type lectins (CD206 and CD205) and the scavenging receptor MARCO. As expected, we observed high levels of activation of macrophages from Sf mice as compared with those from Wt mice (Figure 4B,C). In addition, and unexpectedly, we also observed differential expression in the level of M2 macrophages associated C‐type lectin mannose macrophage receptor CD206^29^ which has been shown to have anti‐inflammatory roles.^30^, ^31^ CD206 was significantly elevated in Wt as compared with Sf macrophages (Figure 4C).

### 3.3 Elevated expression of CTLRs on AMs and pulmonary DCs in the absence of functional T regs

**Figure 5:**
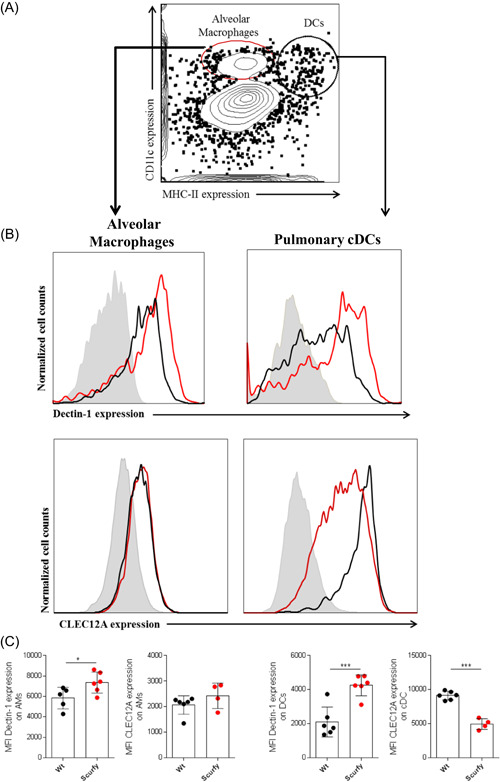
Lack of functional T regs cells affects the expression of C‐type lectin receptors on alveolar macrophages and pulmonary dendritic cells: Flow cytometry‐based identification of alveolar macrophages and dendritic cells in the lungs (A). Histograms depicting relative expression of both Dectin‐1 and CLEC12A on alveolar macrophages (AMs) and lung conventional DCs (cDC) in the lungs of Wt and Sf mice (B). Bar graphs showing quantitative expression of Dectin‐1 and CLEC12A on AMs and cDC (C). All bars display mean ± SEM with *P < .05, and ***P < .001 in two independent experiments with n = 4 to 6 animals/group. DCs, dendritic cells; Dectin‐1, dendritic cell‐associated C‐type lectin‐1; SEM, standard error of mean; T regs, regulatory T cells

To investigate whether the observed differential expression of CTLRs in peritoneal macrophages also occurred in other immune cells involved in antigen presentation, we analyzed the expression of CLEC‐7A (Dectin‐1) and CLEC‐12A (myeloid inhibitory C‐type lectin‐like receptor [MICL]) on AMs and conventional DCs in the lungs of Wt and Sf mice (Figure 5A). Although studies have shown that the CTLR “dendritic cell‐associated C‐type lectin‐1 (Dectin‐1)” is upregulated by proinflammatory conditions and leads to the production of Th1 and/or Th17 immune responses,^32^, ^33^ in case of sterile inflammation, Dectin‐1 has been reported to have an inhibitory function.^34^ Both Dectin‐1 and CLEC12A (MICL) have thus been described to be able to locally suppress myeloid cell activation and promote immune evasion.^24^, ^35^ Moreover, MICL has been shown to be downregulated by proinflammatory stimuli and proposed to have a role in controlling homeostasis and self‐tolerance.^36^ We thus assessed expression patterns of Dectin‐1 and CLEC12A on both AMs and pulmonary conventional DCs in Wt and Sf mice (Figure 5B,C). Taken together, in addition to attenuation of expression levels of various glycosphingolipids on splenic DCs, our data on macrophages and DCs show that whereas CTLRs associated with anti‐inflammatory response such as CLEC12A and CD206 are downregulated in functional T regs lacking Sf mice, other glycan‐binding lectins such as Dectin‐1 and CD205 remain highly expressed in these animals.

## 4 DISCUSSION

With a prediction of about 50% to 60% of the human proteome assumed to be glycosylated, glycosylation is currently accepted as one of the most common and complex types of posttranslational modification.^37^, ^38^ Despite this overwhelming level of posttranslational modification, little is known regarding immunological consequences related to either abundance or patterns of glycosylation on immune cells on homeostatic, inflammatory, and tolerogenic conditions. This lack of information is not only due to the complexicity of glycan structures decorating cell surfaces, levels of glycosylation regulating enzymes, and differential cellular environments but also due to the fact that unlike proteins, glycans are nontemplated.^2^, ^39^, ^40^ Thus, it is a general consensus among glycobiologists that, glycosylation can best be understood in a contextual framework.^2^

Sf mice have an impaired peripheral tolerance characterized by lethal inflammatory multiorgan failure^41^, ^42^ and as such could serve as a model to depict sterile inflammation which can be compared with health. FoxP3 is the master transcription factor required for development towards T regs lineage which through their effector cytokines transforming growth factor‐β and interleukin‐10 suppress inflammation and promote airway remodeling in airway diseases such as allergic asthma. In this study, we speculated the influence of inflammation as observed in scurfy mice would alter glycosylation and specifically glycosphingolipids pattern on immune cells. To address this question, we adopted a high throughput xCGE‐LIF approach and show that, splenic DC from Sf mice lacking functional T regs express lower levels of distinct glycosphingolipids as compared with their Wt counterparts. In addition, we also observed that glycosphingolipids found to be reduced on DCs showed no differential expression on CD4^+^ T cells of Wt and Sf mice. To our knowledge, this is the first study showing differential expression patterns of glycosphingolipids on immune cells due to a lack of functional T reg cells. Although the inflammatory environment as seen in Sf mice would be expected to have a considerable influence on glycosyltransferase and glycosidase activities and epigenetical changes^43^ that would impact the dynamic glycosylation process, information on glycosphingolipids patterns in inflammation and health is still rare.

Despite its exploratory nature, our study raises the question of determinants of glycosphingolipids regulation and function in health vs inflammation as depicted in Sf mice. Previous studies have shown that glycosylation determines the abundance of ligands for lectins which are glycan‐binding proteins.^44^ Indeed, our analysis of selected CTLRs revealed differential expression patterns of CTLRs in Wt compared with Sf mice in that, CTLRs with anti‐inflammatory capacities such as CD206 and CLEC12A were reduced on antigen‐presenting cells of Sf mice. We think this observation provides circumstantial evidence that, availability of distinct glycosphingolipids on surfaces of antigen‐presenting cells such as DCs and macrophages in part determines expression levels of their cognate receptors involved in immune modulation. Thus, our work adds on already available data indicating the influential role of glycosylation levels and glycan‐binding receptors in the regulation of immune responses in inflammation, homeostasis, and tolerance. Human immune deficiencies due to defects in glycan‐binding proteins such as the rare congenital disorder leukocyte adhesion deficiency type 2 or defects leading to reduced expression of Dectin‐1^45^, ^46^, ^47^ have already paved the way in this context. More recent studies linking glycosylation patterns, for example, on immunoglobulins to both severity of inflammatory (auto)immune disorders as well as to protection against viral infections on one hand, and to immunosuppression and induction of tolerance to conditions such as allergic asthma on the other,^48^, ^49^, ^50^, ^51^ confirm the influential role of glycosylation in the regulation of immune responses.

Finally, although we have not addressed the exact mechanisms, we consider the expression pattern and quantities of glycosphingolipids on immune cells to be a valuable target for mapping signatures associated with inflammation and homeostatic conditions in health and thereby, eventually serve as biomarkers for identification of homeostatic perturbances resulting from a diseased condition.

## CONFLICT OF INTERESTS

All the authors declare that there are no conflict of interests.

## AUTHOR CONTRIBUTIONS

ACJ. and GH designed the experiments. ACJ, CR, RG, CH, and FB conducted experiments or helped with analysis. ACJ wrote and GH, CR, RG, CH, RG‐S, and FB corrected or edited the manuscript. All authors read and approved the manuscript.

## Data Availability

The data that support the findings of this study is available from the corresponding author upon reasonable request.

## References

[1] Ohtsubo K , Marth JD . Glycosylation in cellular mechanisms of health and disease. Cell. 2006;126:855‐867. 16959566 10.1016/j.cell.2006.08.019

[2] Baum LG , Cobb BA . The direct and indirect effects of glycans on immune function. Glycobiology. 2017;27:619‐624. 28460052 10.1093/glycob/cwx036

[3] Daniels MA , Hogquist KA , Jameson SC . Sweet 'n' sour: the impact of differential glycosylation on T cell responses. Nat Immunol. 2002;3:903‐910. 12352967 10.1038/ni1002-903

[4] Varki A , Gagneux P . Biological Functions of Glycans In: Varki A, Cummings RD, Esko JD, Stanley P, Hart GW, Aebi M, Darvill AG, Kinoshita T, Packer NH, Prestegard JH, Schnaar RL, Seeberger PH, eds. Essentials of Glycobiology. New York, NY: Cold Spring Harbor; 2015:77‐88. 28876862

[5] Varki A . Biological roles of glycans. Glycobiology. 2017;27:3‐49. 27558841 10.1093/glycob/cww086 PMC5884436

[6] Bax M , Garcia‐Vallejo JJ , Jang‐Lee J , et al. Dendritic cell maturation results in pronounced changes in glycan expression affecting recognition by siglecs and galectins. J Immunol. 2007;179:8216‐8224. 18056365 10.4049/jimmunol.179.12.8216

[7] Comelli EM , Sutton‐Smith M , Yan Q , et al. Activation of murine CD4+and CD8+T lymphocytes leads to dramatic remodeling of N‐linked glycans. J Immunol. 2006;177:2431‐2440. 16888005 10.4049/jimmunol.177.4.2431

[8] Morgan R , Gao G , Pawling J , Dennis JW , Demetriou M , Li B . N‐acetylglucosaminyltransferase V (Mgat5)‐mediated N‐glycosylation negatively regulates Th1 cytokine production by T cells. J Immunol. 2004;173:7200‐7208. 15585841 10.4049/jimmunol.173.12.7200

[9] Freeze HH . Genetic defects in the human glycome. Nat Rev Genet. 2006;7:537‐551. 16755287 10.1038/nrg1894

[10] Lowe JB , Marth JD . A genetic approach to mammalian glycan function. Annu Rev Biochem. 2003;72:643‐691. 12676797 10.1146/annurev.biochem.72.121801.161809

[11] Demetriou M , Granovsky M , Quaggin S , Dennis JW . Negative regulation of T‐cell activation and autoimmunity by Mgat5 N‐glycosylation. Nature. 2001;409:733‐739. 11217864 10.1038/35055582

[12] Lalazar G , Preston S , Zigmond E , Ben Yaacov A , Ilan Y . Glycolipids as immune modulatory tools. Mini Rev Med Chem. 2006;6:1249‐1253. 17100636 10.2174/138955706778742722

[13] Yoshiga Y , Goto D , Segawa S , et al. Activation of natural killer T cells by alpha‐carba‐GalCer (RCAI‐56), a novel synthetic glycolipid ligand, suppresses murine collagen‐induced arthritis. Clin Exp Immunol. 2011;164:236‐247. 21391989 10.1111/j.1365-2249.2011.04369.x PMC3087916

[14] Wu D , Fujio M , Wong CH . Glycolipids as immunostimulating agents. Bioorg Med Chem. 2008;16:1073‐1083. 18006319 10.1016/j.bmc.2007.10.026 PMC2279229

[15] Astronomo RD , Lee HK , Scanlan CN , et al. A glycoconjugate antigen based on the recognition motif of a broadly neutralizing human immunodeficiency virus antibody, 2G12, is immunogenic but elicits antibodies unable to bind to the self glycans of gp120. J Virol. 2008;82:6359‐6368. 18434393 10.1128/JVI.00293-08 PMC2447108

[16] Skuljec J , Jirmo AC , Habener A , et al. Absence of regulatory T cells causes phenotypic and functional switch in murine peritoneal macrophages. Front Immunol. 2018;9:2458. 30429849 10.3389/fimmu.2018.02458 PMC6220442

[17] Rossdam C , Konze SA , Oberbeck A , et al. Approach for profiling of glycosphingolipid glycosylation by multiplexed capillary gel electrophoresis coupled to laser‐induced fluorescence detection to identify cell‐surface markers of human pluripotent stem cells and derived cardiomyocytes. Anal Chem. 2019;91:6413‐6418. 31058489 10.1021/acs.analchem.9b01114

[18] Delmotte P , Degroote S , Lafitte JJ , Lamblin G , Perini JM , Roussel P . Tumor necrosis factor alpha increases the expression of glycosyltransferases and sulfotransferases responsible for the biosynthesis of sialylated and/or sulfated Lewis x epitopes in the human bronchial mucosa. J Biol Chem. 2002;277:424‐431. 11679593 10.1074/jbc.M109958200

[19] Van Dijk W , Mackiewicz A . Interleukin‐6‐type cytokine‐induced changes in acute phase protein glycosylation. Ann N Y Acad Sci. 1995;762:319‐330. 7545370 10.1111/j.1749-6632.1995.tb32336.x

[20] Buzas EI , Gyorgy B , Pasztoi M , Jelinek I , Falus A , Gabius HJ . Carbohydrate recognition systems in autoimmunity. Autoimmunity. 2006;39:691‐704. 17178566 10.1080/08916930601061470

[21] Maverakis E , Kim K , Shimoda M , et al. Glycans in the immune system and the altered glycan theory of autoimmunity: a critical review. J Autoimmun. 2015;57:1‐13. 25578468 10.1016/j.jaut.2014.12.002 PMC4340844

[22] van Kooyk Y , Rabinovich GA . Protein‐glycan interactions in the control of innate and adaptive immune responses. Nat Immunol. 2008;9:593‐601. 18490910 10.1038/ni.f.203

[23] van Vliet SJ , Gringhuis SI , Geijtenbeek TB , van Kooyk Y . Regulation of effector T cells by antigen‐presenting cells via interaction of the C‐type lectin MGL with CD45. Nat Immunol. 2006;7:1200‐1208. 16998493 10.1038/ni1390

[24] Chiffoleau E . C‐Type lectin‐like receptors as emerging orchestrators of sterile inflammation represent potential therapeutic targets. Front Immunol. 2018;9:227. 29497419 10.3389/fimmu.2018.00227 PMC5818397

[25] Toscano MA , Bianco GA , Ilarregui JM , et al. Differential glycosylation of TH1, TH2 and TH‐17 effector cells selectively regulates susceptibility to cell death. Nat Immunol. 2007;8:825‐834. 17589510 10.1038/ni1482

[26] Rabinovich GA , Toscano MA , Jackson SS , Vasta GR . Functions of cell surface galectin‐glycoprotein lattices. Curr Opin Struct Biol. 2007;17:513‐520. 17950594 10.1016/j.sbi.2007.09.002 PMC2100406

[27] Toscano MA , Commodaro AG , Ilarregui JM , et al. Galectin‐1 suppresses autoimmune retinal disease by promoting concomitant Th2‐ and T regulatory‐mediated anti‐inflammatory responses. J Immunol. 2006;176:6323‐6332. 16670344 10.4049/jimmunol.176.10.6323

[28] Perone MJ , Bertera S , Tawadrous ZS , et al. Dendritic cells expressing transgenic galectin‐1 delay onset of autoimmune diabetes in mice. J Immunol. 2006;177:5278‐5289. 17015713 10.4049/jimmunol.177.8.5278

[29] Jablonski KA , Amici SA , Webb LM , et al. Novel markers to delineate murine M1 and M2 macrophages. PLoS One. 2015;10:e0145342. 26699615 10.1371/journal.pone.0145342 PMC4689374

[30] Nawaz A , Aminuddin A , Kado T , et al. CD206(+) M2‐like macrophages regulate systemic glucose metabolism by inhibiting proliferation of adipocyte progenitors. Nat Commun. 2017;8:286. 28819169 10.1038/s41467-017-00231-1 PMC5561263

[31] Hagert C , Sareila O , Kelkka T , Jalkanen S , Holmdahl R . The macrophage mannose receptor regulate mannan‐induced psoriasis, psoriatic arthritis, and rheumatoid arthritis‐like disease models. Front Immunol. 2018;9:114. 29467756 10.3389/fimmu.2018.00114 PMC5808283

[32] Rogers H , Williams DW , Feng GJ , Lewis MA , Wei XQ . Role of bacterial lipopolysaccharide in enhancing host immune response to *Candida albicans* . Clin Dev Immunol. 2013;2013:320168. 23401696 10.1155/2013/320168 PMC3563236

[33] Willment JA , Lin HH , Reid DM , et al. Dectin‐1 expression and function are enhanced on alternatively activated and GM‐CSF‐treated macrophages and are negatively regulated by IL‐10, dexamethasone, and lipopolysaccharide. J Immunol. 2003;171:4569‐4573. 14568930 10.4049/jimmunol.171.9.4569

[34] Huysamen C , Brown GD . The fungal pattern recognition receptor, Dectin‐1, and the associated cluster of C‐type lectin‐like receptors. FEMS Microbiol Lett. 2009;290:121‐128. 19025564 10.1111/j.1574-6968.2008.01418.x PMC2704933

[35] Neumann K , Castineiras‐Vilarino M , Hockendorf U , et al. Clec12a is an inhibitory receptor for uric acid crystals that regulates inflammation in response to cell death. Immunity. 2014;40:389‐399. 24631154 10.1016/j.immuni.2013.12.015

[36] Pyz E , Huysamen C , Marshall AS , Gordon S , Taylor PR , Brown GD . Characterisation of murine MICL (CLEC12A) and evidence for an endogenous ligand. Eur J Immunol. 2008;38:1157‐1163. 18350551 10.1002/eji.200738057 PMC2430328

[37] Kameyama A , Nakaya S , Ito H , et al. Strategy for simulation of CID spectra of N‐linked oligosaccharides toward glycomics. J Proteome Res. 2006;5:808‐814. 16602687 10.1021/pr0503937

[38] Hagglund P , Bunkenborg J , Elortza F , Jensen ON , Roepstorff P . A new strategy for identification of N‐glycosylated proteins and unambiguous assignment of their glycosylation sites using HILIC enrichment and partial deglycosylation. J Proteome Res. 2004;3:556‐566. 15253437 10.1021/pr034112b

[39] Mkhikian H , Mortales CL , Zhou RW , et al. Golgi self‐correction generates bioequivalent glycans to preserve cellular homeostasis. eLife. 2016;5. 10.7554/eLife.14814 PMC4940165 27269286

[40] Jones MB , Oswald DM , Joshi S , Whiteheart SW , Orlando R , Cobb BA . B‐cell‐independent sialylation of IgG. Proc Natl Acad Sci USA. 2016;113:7207‐7212. 27303031 10.1073/pnas.1523968113 PMC4932940

[41] Brunkow ME , Jeffery EW , Hjerrild KA , et al. Disruption of a new forkhead/winged‐helix protein, scurfin, results in the fatal lymphoproliferative disorder of the scurfy mouse. Nat Genet. 2001;27:68‐73. 11138001 10.1038/83784

[42] Bennett CL , Christie J , Ramsdell F , et al. The immune dysregulation, polyendocrinopathy, enteropathy, X‐linked syndrome (IPEX) is caused by mutations of FOXP3. Nat Genet. 2001;27:20‐21. 11137993 10.1038/83713

[43] Potaczek DP , Harb H , Michel S , Alhamwe BA , Renz H , Tost J . Epigenetics and allergy: from basic mechanisms to clinical applications. Epigenomics. 2017;9:539‐571. 28322581 10.2217/epi-2016-0162

[44] Sharon N , Lis H . History of lectins: from hemagglutinins to biological recognition molecules. Glycobiology. 2004;14:53R‐62R. 10.1093/glycob/cwh122 15229195

[45] van de Vijver E , Maddalena A , Sanal O , et al. Hematologically important mutations: leukocyte adhesion deficiency (first update). Blood Cells Mol Dis. 2012;48:53‐61. 22134107 10.1016/j.bcmd.2011.10.004 PMC4539347

[46] Glocker EO , Hennigs A , Nabavi M , et al. A homozygous CARD9 mutation in a family with susceptibility to fungal infections. N Engl J Med. 2009;361:1727‐1735. 19864672 10.1056/NEJMoa0810719 PMC2793117

[47] Ferwerda B , Ferwerda G , Plantinga TS , et al. Human dectin‐1 deficiency and mucocutaneous fungal infections. N Engl J Med. 2009;361:1760‐1767. 19864674 10.1056/NEJMoa0901053 PMC2773015

[48] Alter G , Ottenhoff THM , Joosten SA . Antibody glycosylation in inflammation, disease and vaccination. Semin Immunol. 2018;39:102‐110. 29903548 10.1016/j.smim.2018.05.003 PMC8731230

[49] Kaneko Y , Nimmerjahn F , Ravetch JV . Anti‐inflammatory activity of immunoglobulin G resulting from Fc sialylation. Science. 2006;313:670‐673. 16888140 10.1126/science.1129594

[50] Oefner CM , Winkler A , Hess C , et al. Tolerance induction with T cell‐dependent protein antigens induces regulatory sialylated IgGs. J Allergy Clin Immunol. 2012;129:1647‐1655. 22502800 10.1016/j.jaci.2012.02.037

[51] Epp A , Hobusch J , Bartsch YC , et al. Sialylation of IgG antibodies inhibits IgG‐mediated allergic reactions. J Allergy Clin Immunol. 2018;141:399‐402. 28728998 10.1016/j.jaci.2017.06.021 PMC5758435

